# Hyper-crosslinked β-cyclodextrin porous polymer: an adsorption-facilitated molecular catalyst support for transformation of water-soluble aromatic molecules†
†Electronic supplementary information (ESI) available: Including solid state NMR, TGA, N_2_-adsorption isotherm, XRD, TEM, and adsorption dynamic of aromatic molecules. See DOI: 10.1039/c5sc04034e


**DOI:** 10.1039/c5sc04034e

**Published:** 2015-11-13

**Authors:** Haiying Li, Bo Meng, Song-Hai Chai, Honglai Liu, Sheng Dai

**Affiliations:** a State Key Laboratory of Chemical Engineering and Department of Chemistry , East China University of Science and Technology , Shanghai , 200237 , China . Email: hlliu@ecust.edu.cn; b Chemical Sciences Division , Oak Ridge National Laboratory , Oak Ridge , Tennessee 37831 , USA . Email: dais@ornl.gov; c Department of Chemistry , University of Tennessee , Knoxville , Tennessee 37996 , USA . Email: schai@utk.edu

## Abstract

A hyper-crosslinked β-cyclodextrin porous polymer (BnCD-HCPP) was designed and synthesized facilely by β-cyclodextrin benzylation and subsequent crosslinking *via* a Friedel–Crafts alkylation route. The BnCD-HCPP shows an extremely high BET surface area, large pore volume, and high thermal stability, making it a highly efficient adsorbent for removal of aromatic pollutants from water. The adsorption efficiency in terms of distribution coefficient, defined as the ratio of adsorption capacity to equilibrium adsorbate concentration, ranged from 10^3^ to 10^6^ mL g^–1^ within a concentration of 0–100 ppm, one order of magnitude higher than that of other β-cyclodextrin-based adsorbents reported previously. The molar percentage of adsorbate to β-cyclodextrin exceeded 300%, suggesting that the adsorption occurred not only in the cyclodextrin cavities *via* a 1 : 1 complexation, but also in the nanopores of the BnCD-HCPP created during the hyper-crosslinking. The BnCD-HCPP can be further functionalized by incorporation of gold nanoparticles for catalytic transformation of adsorbed phenolic compounds such as 4-nitrophenol to 4-aminophenol.

## Abstract 2

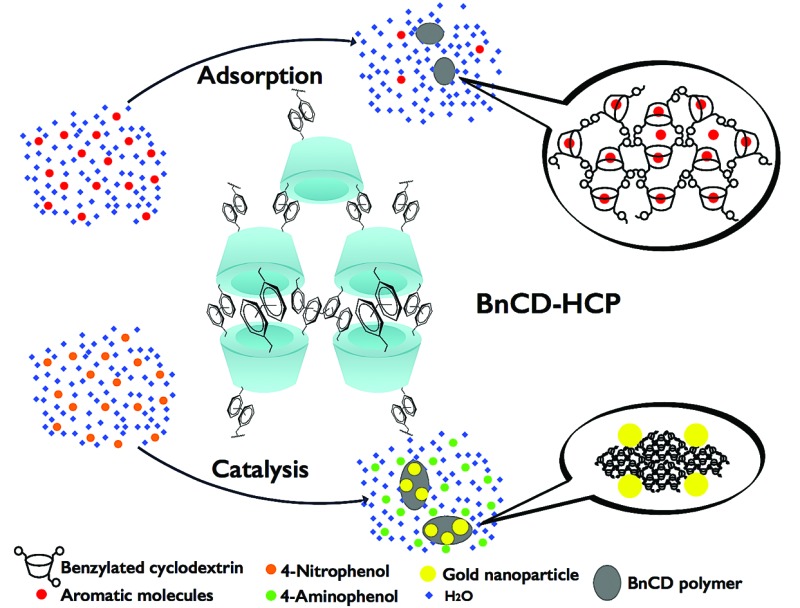
A hyper-crosslinked β-cyclodextrin porous polymer (BnCD-HCPP) was designed and synthesized facilely by β-cyclodextrin benzylation and subsequent crosslinking for efficient adsorption and catalysis.

## Introduction

β-Cyclodextrin (β-CD), consisting of seven α-linked d-glucopyranose units, is a unique cyclic oligosaccharide with a hydrophobic center and hydrophilic edge. Based on its chiral cavity, β-CD shows an exceptional ability to selectively bind nonpolar suitably sized aliphatic and aromatic molecules to form inclusion complexes, demonstrating great potential in various applications such as catalysis, adsorption, and separation of organic pollutants from contaminated water.^1^ The high solubility of β-CD in water, however, impedes its practical application in aqueous systems, motivating researchers to develop a water-insoluble β-CD in a solid state.^2^ Two general strategies have been explored to date: (1) direct polymerization and/or crosslinking of β-CD using a coupling agent such as epichlorohydrin (EPI) (CD-polymers)^3^ and (2) attachment of β-CD *via* chemical linkers to water-insoluble supporting materials (CD-coated/incorporated materials).^4,5^

Traditional CD-polymers, which are crosslinked directly by toxic EPI and other small linkers, have little framework nanoporosity except inherent cavities of cyclodextrin, leading to poor specific surface area (<100 m^2^ g^–1^).^2,3^ This drawback limits the access of CD cavities to adsorbate molecules, significantly reducing the adsorption capacity and efficiency of the CD polymers. In addition, their low thermal and chemical stabilities curtail their large-scale application. To overcome these disadvantages, CDs are chemically grafted onto the exterior surfaces of amorphous polymers, silica gels, and nanoparticles (CD-coated materials),^4^ or onto the internal surfaces of inorganic/organic porous materials with uniform pore structure and high surface area (CD-incorporated materials, *e.g.*, CD-HMS,^5a–c^ CD-nanofiber^5*d*^). These supporting materials help achieve rapid adsorption, by providing easy access to binding sites, and enhance the thermal stability of grafted CDs. However, the CD-coated/incorporated materials are still subject to relatively low surface area after the grafting of β-CD, limiting their adsorption capacities for organic pollutants. Hence, a direct synthesis of CD-containing polymers with high surface area and thermal stability is highly desirable but still remains a challenge.

In this effort, we aimed to directly crosslink β-CD *via* a simple Friedel–Crafts alkylation route. Generally, hyper-crosslinked polymers created by facile Friedel–Crafts alkylation, which have been widely applied in gas storage and separation, show high surface area and good thermal stability but require benzyl structure units for alkylation and skeleton support.^6^ Bearing this in mind, we first synthesized fully benzylated β-CD (BnCD) and then hyper-crosslinked it with formaldehyde dimethyl acetal (FDA) as an external cross-linker and FeCl_3_ as a Lewis acid catalyst. The resulting BnCD-based hyper-crosslinked porous polymer (BnCD-HCPP) indeed showed high surface area and thermal stability, making it an exceptional adsorbent for aromatic compounds, as well as a support for gold catalysts for further transformation of the adsorbed aromatic molecules.

## Results and discussion

**Scheme 1:**
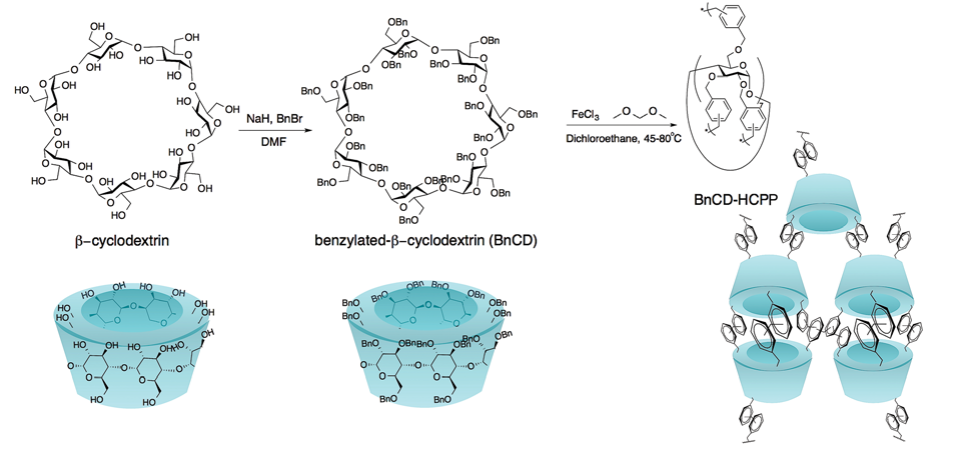
Synthetic strategy of the hyper-crosslinked β-cyclodextrin porous polymer (BnCD-HCPP).

**Fig. 1:**
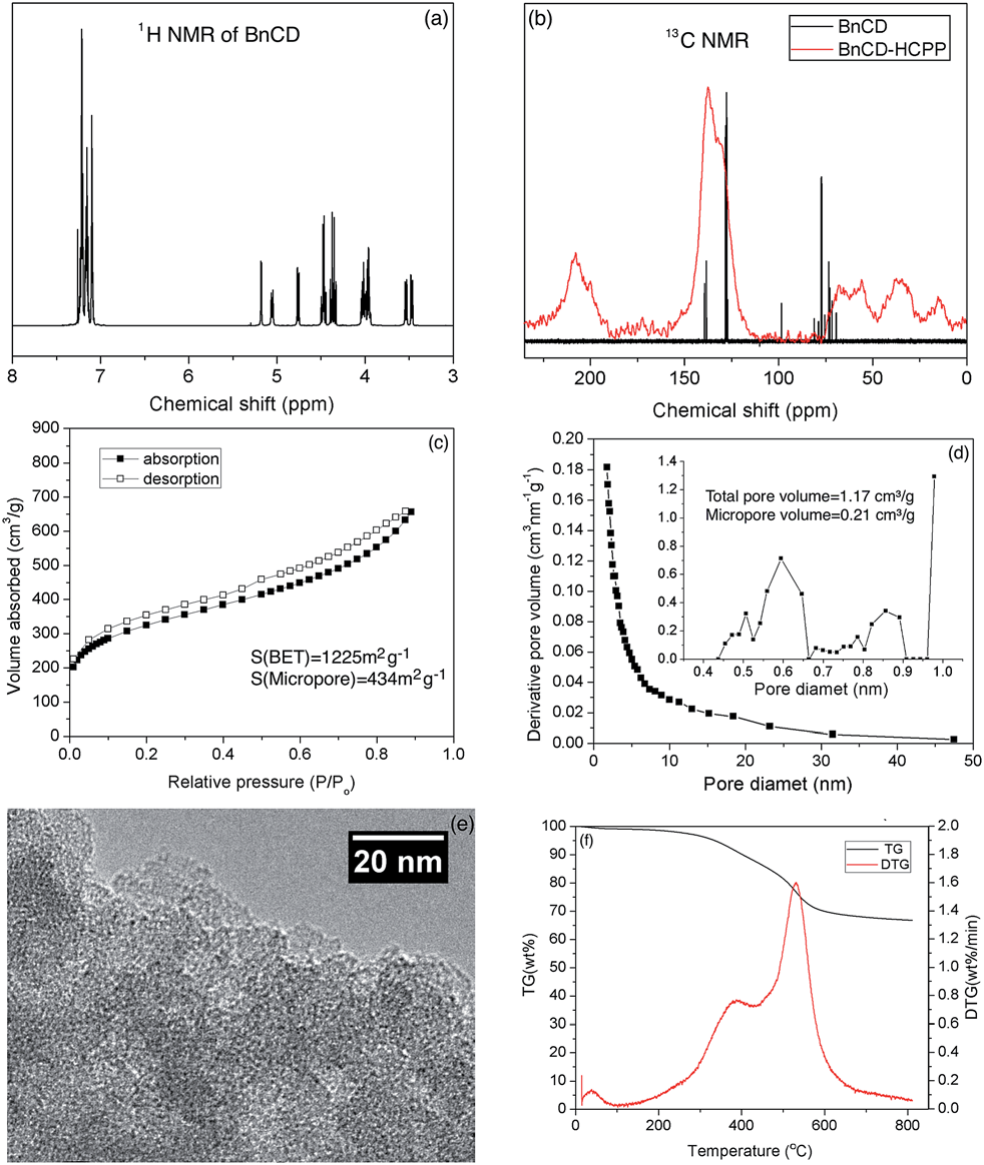
(a) ^1^H NMR spectrum of BnCD, (b) ^13^C NMR of BnCD and solid-state ^13^C NMR of BnCD-HCPP (spinning at 7 K), (c) N_2_ adsorption–desorption isotherms of BnCD-HCPP measured at 77 K (the polymer was pretreated at 170 °C under nitrogen flow for 2 h), (d) pore size distribution of BnCD-HCPP, (e) transmission electron microscope image of BnCD-HCPP, and (f) thermogravimetry and differential thermogravimetry curves of BnCD-HCPP.

As shown in Scheme 1, benzylation of β-CD was carried out with NaH and benzyl bromide in dry DMF at 0 °C. After stirring overnight at room temperature, the reaction mixture was concentrated and extracted with methylene chloride and finally purified by silica gel chromatography for a total yield of 83%.^7^ A complete benzylation of all the hydroxyl groups on β-CD was confirmed by ^1^H nuclear magnetic resonance (NMR) and ^13^C NMR (Fig. 1a and b). The peaks between 7.0 and 7.5 ppm in ^1^H NMR and the peaks between 120 and 140 ppm in ^13^C NMR are attributed to three substituted phenyl groups on C2, C3, and C6 hydroxyl groups; and the remaining peaks correspond to the carbohydrate backbone and the methylene group on the benzyl group. The resultant BnCD was then hyper-crosslinked *via* oxidative coupling polymerization, by adding FDA and anhydrous FeCl_3_ in dry C_2_H_4_Cl_2_ under a nitrogen atmosphere. After polymerization at 80 °C for 24 h, the obtained brown polymer was washed in methanol and water a few times and then purified by Soxhlet extraction with methanol.^8^ The chemical structure of BnCD-HCPP was characterized by solid-state ^13^C CP/MAS NMR (Fig. 1b). Clear resonance peaks appeared around 130 and 40–50 ppm, corresponding to aromatic carbons and linkers. The peak at 60–70 ppm is related to the skeleton of β-CD in the polymer.

Notably, the synthesized BnCD-HCPP bore a BET surface area as high as 1225 m^2^ g^–1^ (micropore surface area of 434 m^2^ g^–1^) and a total pore volume of 1.71 cm^3^ g^–1^, as shown in Fig. 1c and d. The high surface area could be a result of not only the inherent cavitation of cyclodextrin at the subnanometer level (Fig. 1e) but also, more significantly, inefficient packing of rigid and contorted benzene rings. In addition, based on thermal-gravimetric analysis (TGA) (Fig. 1f), this BnCD-HCPP polymer remained at over 67 wt% even at 800 °C under nitrogen and showed almost no obvious weight loss until 250 °C, showing extraordinary thermal stability compared with other CD-containing solid materials.

**Fig. 2:**
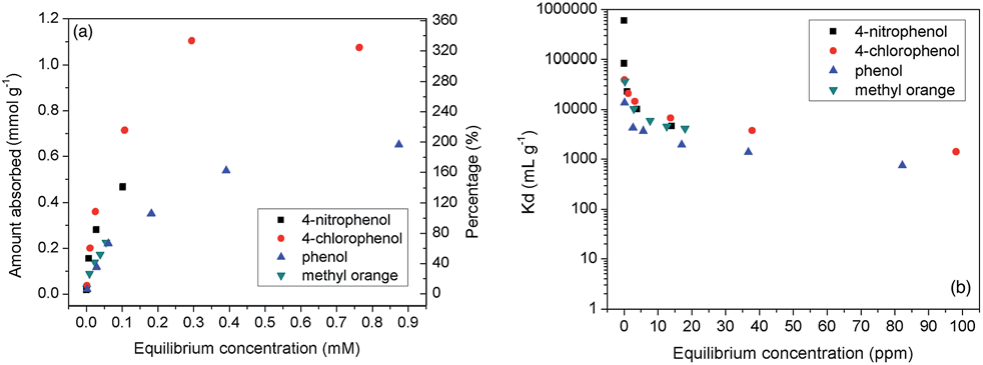
Adsorption isotherms (a) and distribution coefficient (b) of BnCD-HCPP toward *p*-nitrophenol, *p*-chlorophenol, phenol, and methyl orange. The percentage in (a) was calculated as a molar percentage (mol/mol%) of the absorption amount (mmol g^–1^) to the amount of CD units (mmol g^–1^) in the adsorbent.

It is interesting that the BnCD-HCPP showed great efficiency in removing aromatic molecules from an aqueous solution. Three typical aromatic molecules (4-nitrophenol, 4-chlorophenol, phenol) and one representative dye molecule (methyl orange) were chosen as the adsorbates. 5 mg of the polymer was dipped into 25 mL of 4-nitrophenol, 4-chlorophenol, and phenol aqueous solutions of different concentrations and shaken for 24 h to obtain adsorption isotherms (Fig. 2). To evaluate the adsorption efficiency of the aromatic reagent removal from water by BnCD-HCPP, the distribution coefficient of the adsorbent, *K*_d_, was calculated^5*a*^ by eqn (1):

1 *K*_d_ = (*C*_i_ – *C*_f_)*V*/(*C*_f_*m*),

where *C*_i_ and *C*_f_ are the concentrations of the initial solution and the solution after adsorption, respectively; *V* is the volume of the solution (mL); and *m* is the mass of the adsorbent (g). As illustrated in Fig. 2, the adsorption capacity for 4-chlorophenol was up to 1.10 mmol g^–1^ at the equilibrium concentration of 0.7 mmol L^–1^; and the values of *K*_d_ for the BnCD-HCPP were in the range of 10^3^–10^6^ mL g^–1^, much higher than those of other β-cyclodextrin-based adsorbents reported previously (Table S1†).^9^ These values suggest BnCD-HCPP to be a highly effective adsorbent for aromatic molecule removal. Overall, the *K*_d_ values decreased with an increase in the equilibrium concentration of the adsorbates, especially in the lower concentration range. This can be ascribed to the strong binding interactions between the targeted aromatic rings and the specific binding sites in the adsorbent, namely, the cyclodextrin rings in the BnCD-HCPP.^10^ If the initial concentration of the solution is increased, the most effective binding sites in the polymer, the cyclodextrin cavities, will be occupied until saturation, leading to a decrease in the binding affinity of the adsorbent. This reveals that the cyclodextrin rings play a critical role in this adsorption process. However, the function of the polymer nanopores created during the hyper-crosslinking cannot be ignored. The maximum capacities of these adsorbate molecules exceeded the amount of CD groups in the adsorbent (up to 330 mol%, Fig. 2a), indicating that the adsorption occurred not only in the cyclodextrin region, *via* the formation of a 1 : 1 complexation,^2,5a^ but also in the nanopores in the BnCD-HCPP. In addition, the complete adsorption of phenol from water by BnCD-HCPP took about 60 min, whereas the adsorption of the other aromatic molecules required more time because of their higher capacities (Fig. S1†). The cyclic stability of the polymer was also investigated by performing the same adsorption process with the same adsorbent four times. After each use, the polymer was recycled by centrifugation, washed with ethanol, and dried overnight. The recycled BnCD-HCPP can still adsorb over 90% as much of the 4-nitrophenol as it adsorbed the first time (Fig. S2†).

The great efficiency of BnCD-HCPP in adsorbing 4-nitrophenol from water inspired us to use BnCD-HCPP as a supporting material for a gold catalyst; the 4-nitrophenol reduction reaction was chosen to investigate the catalytic activity of the synthesized Au@BnCD-HCPP. The BnCD-HCPP was added into a gold(iii) chloride aqueous solution after the pH was adjusted to 10. After the solution was heated for 1 h in a water bath, the precipitate was collected by centrifugation, washing by water, and drying overnight in a vacuum oven. The existence of gold particles in the polymer was confirmed by an X-ray diffraction spectrum with a peak at 2*θ* = 38° (Fig. S3†). Gold nanoparticles with an average diameter of 8.4 nm were spread on the polymer support shown in the transmission electron microscope images (Fig. S4†).

**Fig. 3:**
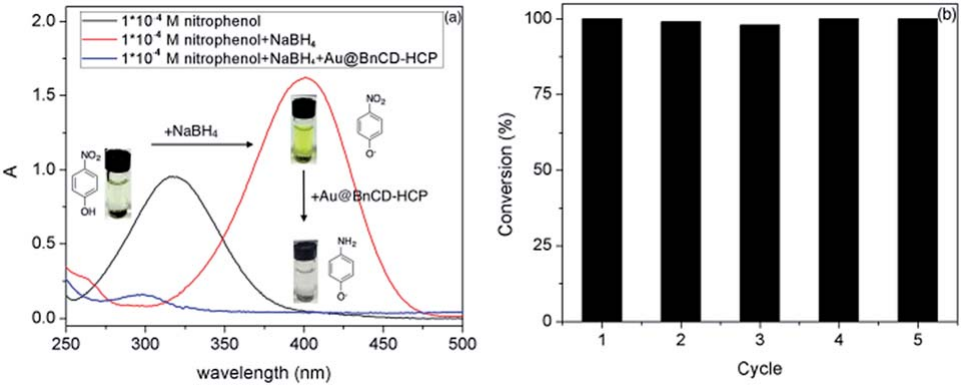
Ultraviolet-visible spectra of 4-nitrophenol before and after catalytic reduction by Au@BnCD-HCPP (a) and the catalyst recycling test (b).

The reduction of 4-nitrophenol to 4-aminophenol was completed in only 3 min after the addition of a catalytic amount (5 mg) of Au@BnCD-HCPP catalyst under stirring at room temperature. As shown in Fig. 3, the light yellow 4-nitrophenol solution with an absorption peak at 318 nm turned bright yellow with a peak at 400 nm after an NaBH_4_ solution was added. When the Au@BnCD-HCPP catalyst was added, the solution quickly became colorless and the absorption peak at 400 nm decreased, while the peak at 295 nm simultaneously increased. The rapid reaction of 4-nitrophenol is related to facile diffusion of the reactant through the pores in the BnCD-HCPP of high surface area.^11–13^ The great affinity of the BnCD-HCPP for the reactant may enrich the reactant on the surface of the catalyst compared with the bulk solution, consequently increasing the reaction rate.^14^ The stability of the catalyst was also examined by applying the same catalyst into the reduction reaction five times. The catalyst was simply isolated by centrifugation, washed with deionized water, and dried overnight after each use. In each reaction cycle, the catalyst was still highly effective in reducing 4-nitrophenol by nearly 100% conversion in 3 min. Apparently, the existence of porous BnCD-HCPP can help stabilize gold particles from aggregation and facilitate the diffusion of reactants, which makes it a promising catalyst of high reactivity and stability in large-scale applications.

## Conclusions

In conclusion, we have developed a new route to synthesizing water-insoluble cyclodextrin polymers of high porosity and great thermal stability by simple benzylation and Friedel–Crafts alkylation polymerization. This hyper-crosslinked porous polymer based on cyclodextrin (BnCD-HCPP) shows great efficiency in the adsorption of aromatic molecules from water and also can serve as an excellent support for gold nanoparticles for further catalytic transformation of the adsorbed molecules.

## Experimental sections

### Benzylated β-CD (BnCD) synthesis

β-Cyclodextrin (500 mg, 0.44 mmol) was dissolved in dry DMF (10 mL). The solution was cooled to 0 °C and added NaH (60%, 740 mg, 18.48 mmol) portionwise. After stirring for 15 min, benzyl bromide (2.20 mL, 18.48 mmol) was slowly added and the reaction mixture was warmed to room temperature. After stirring overnight, the reaction mixture was quenched by adding methanol (5 mL) and concentrated *in vacuo*. The resulting residue was mixed with water (100 mL) and extracted with methylene chloride (3 × 50 mL). The combined organic layers were dried over anhydrous Na_2_SO_4_ and concentrated. The crude product was purified by silica gel chromatography (hexanes : EtOAc = 8 : 1) to yield BnCD (1.10 g, 83%) as colorless oil. ^1^H NMR (CDCl_3_, 600 MHz) *δ* 7.24–7.08 (m, 105H), 5.18 (d, *J* = 3.5 Hz, 7H), 5.06 (d, *J* = 10.9 Hz, 7H), 4.77 (d, *J* = 10.9 Hz, 7H), 4.48 (q, *J* = 12.1 Hz, 14H), 4.37 (q, *J* = 12.1 Hz, 14H), 4.05–3.94 (m, 28H), 3.55 (m, 7H), 3.48 (dd, *J* = 9.5, 3.5 Hz, 7H). ^13^C NMR (CDCl_3_, 151 MHz) *δ* 139.39, 138.46, 138.31, 128.41, 128.27, 128.10, 127.94, 127.65, 127.63, 127.55, 127.40, 127.04, 98.57, 81.02, 78.91, 78.80, 75.54, 73.39, 72.79, 71.62, 69.41. MALDI-TOFMS: calcd for C_189_H_196_O_35_·Na^+^: 3050.3514; found: *m*/*z* 3050.3516. The data of BnCD are consistent with those previously reported.

### BnCD-HCPP polymerization

Typically, to a solution of the BnCD (0.46 g) and FDA (0.48 g) in anhydrous 1,2-dichloroethane (10 mL), anhydrous FeCl_3_ (1.03 g) was slowly added under nitrogen atmosphere. The mixture was then heated to 45 °C for 5 h and 80 °C for 19 h. The resulting brown precipitate was collected and washed with methanol and water until the filtrate became colorless and further purified by Soxhlet extraction with methanol for 24 h. The polymer was dried under vacuum for 24 h at 50 °C.

### Adsorption measurement of aromatic molecules

The aqueous solutions of aromatic molecules (4-nitrophenol, 4-chlorophenol, phenol and methyl orange) with the concentration in the range of (5–1000) × 10^–6^ M (*i.e.*, 5–1000 ppm) were prepared for the adsorption test. BnCD-HCPP (5 mg) was added into the selected solutions (25 mL) and then shaken for 24 h. The solution was separated by centrifugation and the concentration was tested with UV/Vis spectroscopy.

For the dynamic study, BnCD-HCPP (5 mg) was added into the aromatic molecule (4-nitrophenol, 4-chlorophenol, phenol and methyl orange) solutions (1 × 10^–4^ M, 25 mL), which were shaken for specific time periods. A small amount of the solution was taken out by glass pipette for UV/Vis spectroscopy and pulled back after the test.

For the recycle test, BnCD-HCPP (5 mg) was added into the 4-nitrophenol solutions (1 × 10^–4^ M, 25 mL), which were shaken for 24 h. The solution and the absorbent were separated by centrifugation. After each use, the BnCD-HCPP was washed by ethanol and deionized water for several times and dried overnight at 50 °C in the oven.

### Synthesis of gold catalyst on BnCD-HCPP (Au@BnCD-HCPP)

Generally, 8 mg of gold(iii) chloride trihydrate (HAuCl_4_·3H_2_O) was dissolved in 2 mL of deionized water. After adjusting the PH to 10 by adding 0.1 M NaOH aqueous solution, BnCD-HCPP was dipped in. The mixed solution was heated to 80 °C in water bath for 1 h while the PH was controlled at 10. Subsequently, the precipitates were separated by centrifugation and washed with deionized water several times. Finally, the product Au@BnCD-HCPP was dried overnight at 50 °C in the vacuum oven.

### Catalyst test for 4-nitrophenol reduction

Aqueous NaBH_4_ solution (0.3 M, 0.1 mL) was added into 3 mL 4-nitrophenol solution (1 × 10^–4^ M), and 5 mg of Au@BnCD-HCPP was then added under stirring. For the recycle test, the catalyst was separated by centrifugation, washed with deionized water and dried overnight at 50 °C in the oven after each use (3 min of the reaction).
