# Supplementary material for: Hyper-crosslinked β-cyclodextrin porous polymer: an adsorption-facilitated molecular catalyst support for transformation of water-soluble aromatic molecules
Source: Chem Sci. 2015 Nov 13;7(2):905–9. doi: 10.1039/c5sc04034e (PMC5530358; doi:10.1039/c5sc04034e)
Supplement: Supplementary file 1 [file SC-007-C5SC04034E-s001.pdf]

Electronic Supplementary Material (ESI) for Chemical Science.
This journal is © The Royal Society of Chemistry 2015

Supporting Information

Hyper-crosslinked β-Cyclodextrin Porous Polymer: An Adsorption-Facilitated Molecular Catalyst Support for Transformation of Water-Soluble Aromatic Molecules

Haiying Li,[†,‡] Bo Meng,[§] Song-Hai Chai,[*,§] Honglai Liu,[*,†] Sheng Dai[*,‡,§]

[†]State Key Laboratory of Chemical Engineering and Department of Chemistry, East China University of Science and Technology, Shanghai, 200237, China

[‡]Chemical Sciences Division, Oak Ridge National Laboratory, Oak Ridge, Tennessee 37831, United States

[§]Department of Chemistry, University of Tennessee, Knoxville, Tennessee 37996, United States

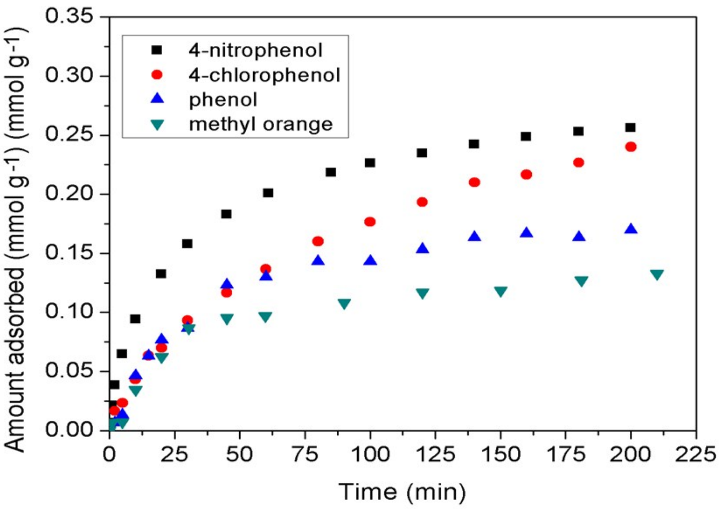

Figure S1. Adsorption amount of aromatic molecules for BnCD-HCP as a function of adsorption time. The initial concentration of 4-nitrophenol, 4-chlorophenol, phenol and methyl orange aqueous solution was 0.1mM.

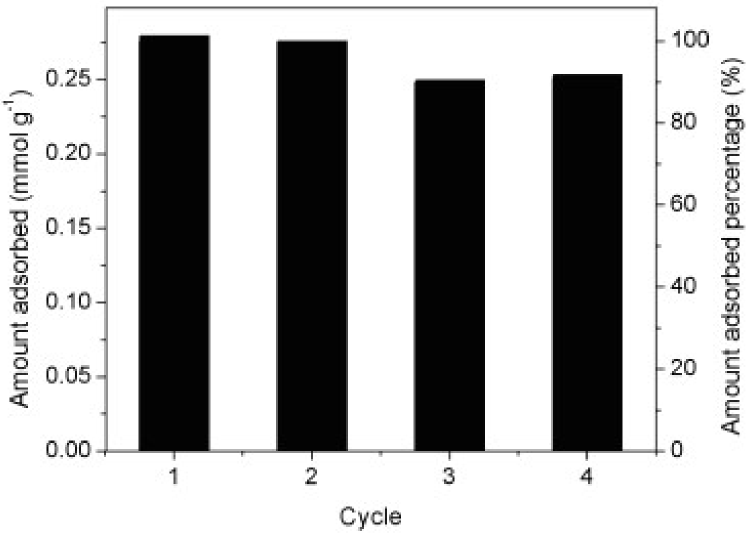

Figure S2. The recycle adsorption tests of a 4-nitrophenol aqueous solution (0.1mM) for BnCD-HCPP. The percentage adsorbed is the adsorbed amount in each cycle divided by that in the first cycle.

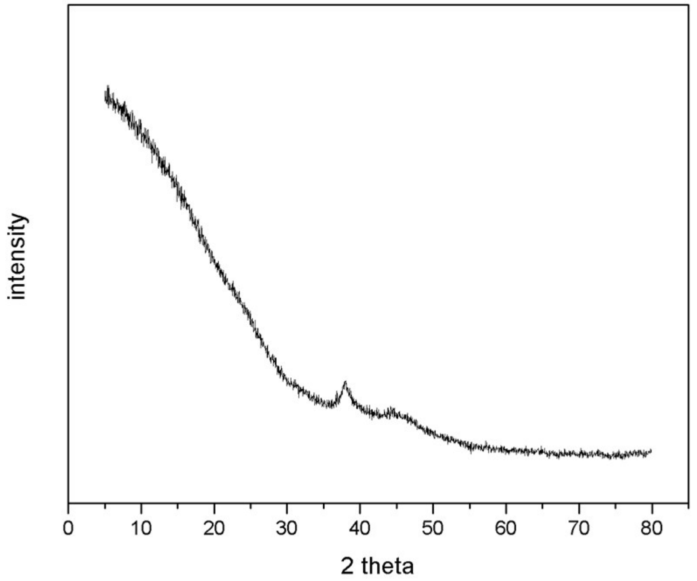

Figure S3. XRD pattern of Au@BnCD-HCPP.

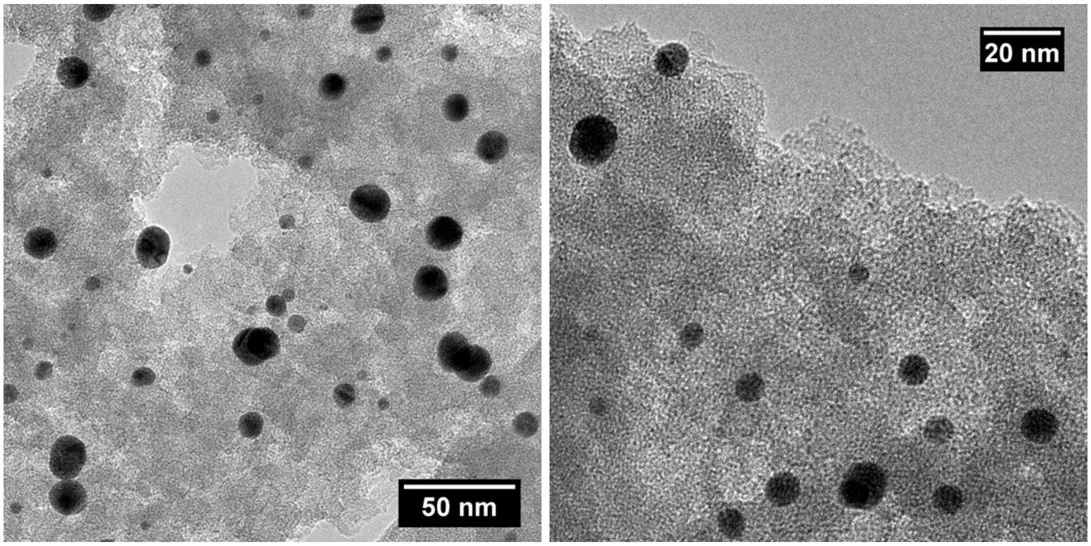

Figure S4. TEM images of Au@ BnCD-HCPP.

Table S1. Comparison of the adsorbed amount (q_e) and distribution coefficients (K_d) for aromatic molecules adsorption from water by different materials. (some K_d values are calculated based on the experimental conditions listed in the reference)

Material	Aromatic molecules	Initial concentration (mmol/L)	Equilibrium concentration (mmol/L)	q_e (mmol/g)	K_d (mL/g)	Ref.
BnCD-HCP	4-nitrophenol	$3*10^{-3}$~0.2	$3*10^{-5}$~0.1	0.02~0.47	$4.6*10^3$~$6.0*10^5$	Our work
	Phenol	$5*10^{-3}$~1	$1.5*10^{-3}$~0.8	0.02~0.65	$7.4*10^2$~$1.3*10^4$	Our work
	4-chlorophenol	$8*10^{-3}$~0.98	$9*10^{-4}$~0.7	0.04~1.10	$1.4*10^3$~$3.9*10^4$	Our work
$Fe_3O_4@SiO_2$-PGMACD	bisphenol-A	0.2	-	0.13	$2.0*10^3$	S1
CD-HMS (8%)	4-nitrophenol	-	0-0.1 [a]	0.36	$1*10^3$~$1*10^4$ [a]	S2
	Phenol	-	0-0.3 [a]	0.15	$1*10^{2.5}$~$1*10^{3.9}$ [a]	S2
	4-chlorophenol	-	0-0.2 [a]	0.18	$1*10^{2.5}$~$1*10^{3.5}$ [a]	S2
SCD-ZnAl LDH	Hydroquinone (for 2 days)	0.5 (mg/mL)	0.19(mg/mL)	0.47	101	S3
	2,3-dimethylphenol (for 2days)	0.5 (mg/mL)	0.04(mg/mL)	0.49	18	S3
beta-CDP (max capacity)	bisphenol A	0.5		0.25	1000	S4
CD-zeolite	p-nitrophenol	0.72 (100mg/L)	-	0.002 (0.25mg/g)	-	S5

a) The data was directly read from the figures in this reference

REFERENCE

[S1] Kang, Yan. et al. β-Cyclodextrin-modified hybrid magnetic nanoparticles for catalysis and adsorption J. Mater. Chem., 2011, 21, 3704-3710

[S2] Bibby, A. & Mercier, L. Adsorption and separation of water-soluble aromatic molecules by cyclodextrin-functionalized mesoporous silica. Green. Chem., 2003, 5, 15-19.

[S3] Xue, X. et al. Nanocage Structure Derived from Sulfonated β-Cyclodextrin Intercalated Layered Double Hydroxides and Selective Adsorption for Phenol Compounds. Inorg. Chem. 2014, 53, 1521−1529

[S4] Hiroyuki Kono,Taichi Nakamura. Polymerization of β-cyclodextrin with 1,2,3,4-butanetetracarboxylic dianhydride: Synthesis, structural characterization, and bisphenol A adsorption capacity. Reactive & Functional Polymers, 2013, 73, 1096–1102

[S5] Li, XH.; Zhu, K.; Hao, XK. Surface modification of zeolite with beta-cyclodextrin for removal of p-nitrophenol form aqueous solution. Water Science and Technology, 2009, 60, 329-337.
